# Pesamosca osteoplasty: surgical procedure for the spatial correction of cubitus varus or valgus post malunited supracondylar fractures of the humerus

**Published:** 2014

**Authors:** G Burnei, Ş Gavriliu, I Nepaliuc, C Vlad, M Drăgoescu, I Georgescu, RA Ghita, L Muntean, AA Pârvan, C Dughilă, I Ţiripa, Ş Hamei, I Klinaku

**Affiliations:** * “Carol Davila” University of Medicine and Pharmacy, Bucharest, Romania; ** “Maria Sklodowska Curie” Children’s Clinical Emergency Hospital, Bucharest, Romania; *** The Studies and Research Group in Pediatric Orthopedics-2012; **** “Nicolae Testemiţeanu” State University of Medicine and Pharmacy, Chisinau, Republic of Moldova; ***** Children’s Clinical Hospital, Brasov, Romania; ****** “Floreasca” Clinical Emergency Hospital, Bucharest, Romania; ******* “St. Spiridon” Clinical Emergency County Hospital, Bucharest, Romania; ******** County Hospital, Tulcea, Romania; ********* Q.K.U. University, Pristina, Kosovo

**Keywords:** cubitus varus/ valgus, malrotation, malunion of the supracondylar fracture, spatial correction, metaphyseal diaphyseal distal humeral osteoplasty

## Abstract

**Introduction.** Supracondylar fractures of the humerus represent a current concern in the child’s and adolescent’s osteo-articular pathology. Even though orthopedic reductions are made correctly, fractures can become displaced when managed only by cast immobilization and complications may arise. The most frequent complications encountered in “Prof. Dr. Alexandru Pesamosca” Clinique, Bucharest, Romania, due to supracondylar humeral fractures, are valgus or varus deviations with angles that can sometimes exceed 40 degrees as a result of malunion.

Varus or valgus deformations were rarely encountered after surgical treatment.

The goal of this study is to present an alternative surgical technique to correct varus and valgus deformations as well as malrotation.

**Material and method.** The study is a retrospective analysis of a 96 children study group surgically managed during 1985 and 2013. In the first period, various surgical techniques have been performed: cuneiform resections, step-cut osteotomies, open wedge osteotomies with external fixation, epiphysiodesis, hemichondrodiatasis and Pesamosca metaphyseal diaphyseal osteoplasty. Starting with 2005, all the cases that presented such complications – 28 out of 96 (29.1%) – were managed with the Pesamosca procedure.

Due to the malunion of supracondylar humeral fractures only varus or valgus deformities were admitted in the study. The malunion due to the pathologic fractures encountered in osteogenesis imperfecta or fibrous dysplasia was precluded. The experience accumulated with the other surgical techniques used in 68 out of 96 patients (70.9%) determined us to exclusively use the Pesamosca osteoplasty following the year 2005, seeing the simplicity and the efficiency of this procedure.

**Results.** The outcome was very good. In 5 cases out of the 28 (17%) an apparent residual elbow was encountered and one case of relapse (3%) was noted due to inadequate term of cast immobilization. The elbow’s mobility was completely recovered, the thoracic member’s axis was appropriate and the metaphyseal diaphyseal osteotomy site healed completely in 3 months’ time.

**Conclusions.** Compared to other surgical techniques, the Pesamosca technique offers to the surgeon the possibility of correcting the varus or the valgus deformity as well as the malrotation in a simple, secure and efficient manner.

## Introduction

During 1970-1990, the number of complications that occurred second to supracondylar fractures of the humerus has risen in Romania. Not being satisfied with the existing techniques used to correct varus or valgus deviations of the elbow, Professor Dr. Alexandru Pesamosca had conceived an original method to treat these complications, by using a “Z” osteotomy and cuneiform resections proximal and distal to the tranches.

After surgically treating two patients in whom he had good results, he presented a paper to the Romanian Medical Science Union, which later became, in our opinion, the safest and the most efficient technique of correcting cubitus varus or valgus. The paper’s title was “Personal correction procedure for the post-traumatic cubitus varus” and, in its content, “A personal procedure for the correction of varus and valgus deformities of the elbow due to supracondylar fractures” is presented. The procedure consists in achieving a Z osteotomy, which consecutively allows the correction of a possible malrotation. The procedure was applied in two cases, with good results”.

At present, The Studies and Research Group in Pediatric Orthopedics retrospectively analyzed the casuistry and restored the steps of surgery, in rendering this procedure to the literature of specialty. The procedure was communicated in the national conferences and congresses held in Cluj, Bucharest, Chisinau and in the 29th annual congress of the European Pediatric Orthopedics Society (EPOS) held in Zagreb on the 7th-10th of April 2010 [**1**-**6**], to be within reach to all the doctors interested in these pathologies.

## Material and method

Varus and valgus deviation of the elbow are unaesthetic and, rarely, they can present functional complications, especially when Baumann’s angle exceeds 40 degrees: limitation in flexion/extension of the elbow, late paralysis of the ulnar nerve in cubitus valgus, elbow instability, iterative fractures.

The Study Group analyzed patients with cubitus varus and valgus operated at “Alexandru Pesamosca” Clinique, M. S. Curie Hospital during 2005 and 2013. During 2000 and 2005, other surgical techniques were performed for the correction of these complications. The Pesamosca osteoplasty was exclusively performed following 2005. During the first period, various operations such as the following were performed: cuneiform resections, step-cut osteotomies, open wedge osteotomies with external fixation, epiphysiodesis, hemichondrodiatasis and Pesamosca metaphyseal diaphyseal osteoplasty. The statistics of the Pediatric Orthopedic Department comprises 96 children operated between 1985 and 2013, who presented with axial deviations of the elbow, varus and valgus, secondary to humeral supracondylar fractures. Starting with 2005, all the patients who presented with these complications, 28 out of 96 (29.1%), were operated by using the Pesamosca procedure.

**Surgical technique**

We exhibited this technique, given the fact that it is easy to perform technique in every pediatric orthopedics department.

**Fig. 1:**
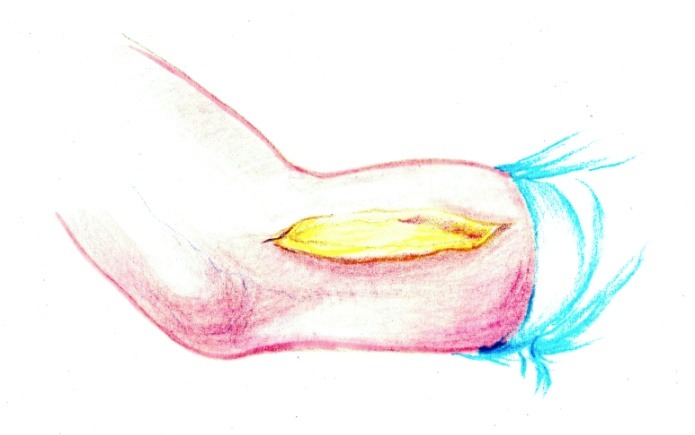
Approximately 10 to 12 cm incision

**Fig. 2:**
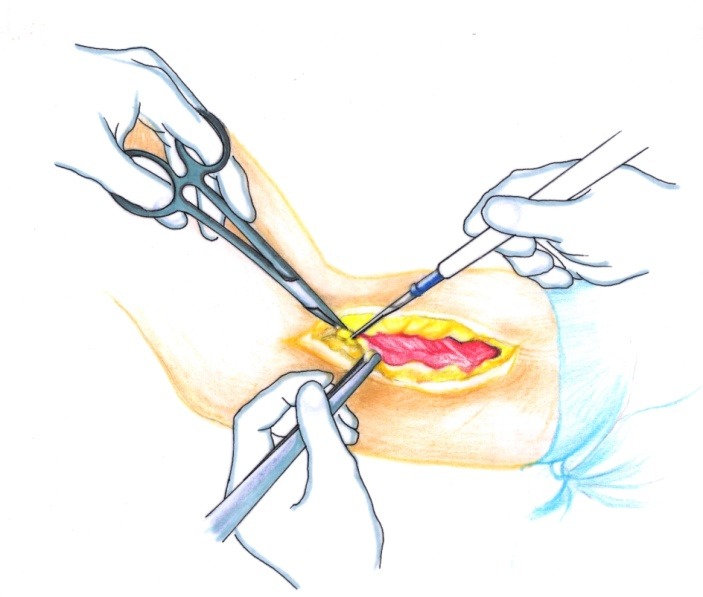
The brachial fascia is sectioned where the lateral intermuscular septum is reflected

**Fig. 3:**
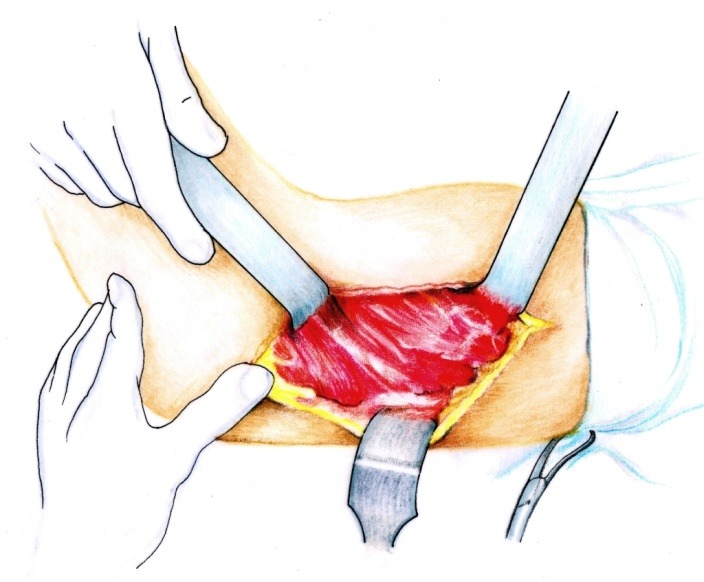
The brachial muscle is disinserted from the septum

**Fig. 4:**
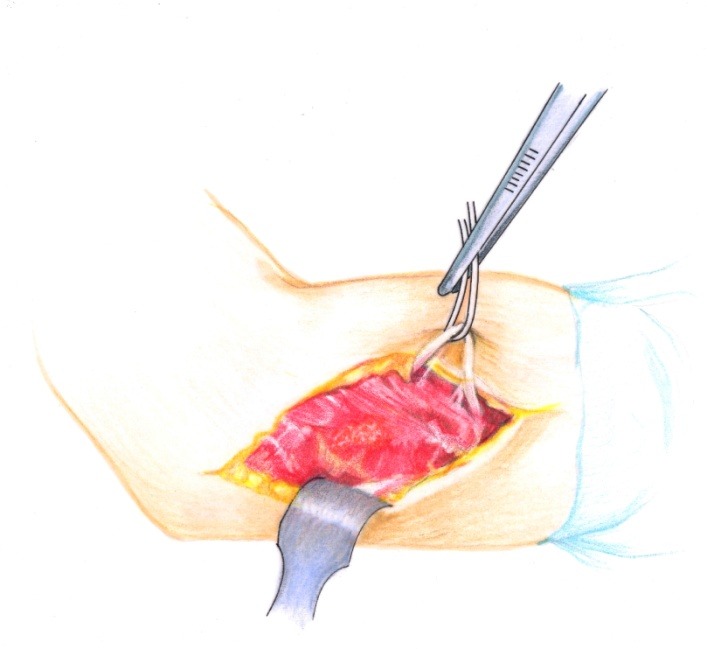
The radial nerve is isolated on a cord in order to avoid damage to this structure during the osteoplasty

**Fig. 5:**
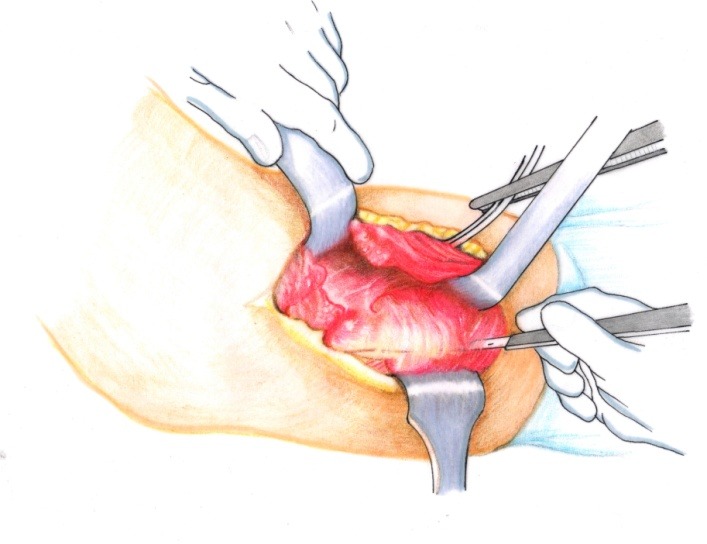
The periosteum is incised longitudinally

**Fig. 6:**
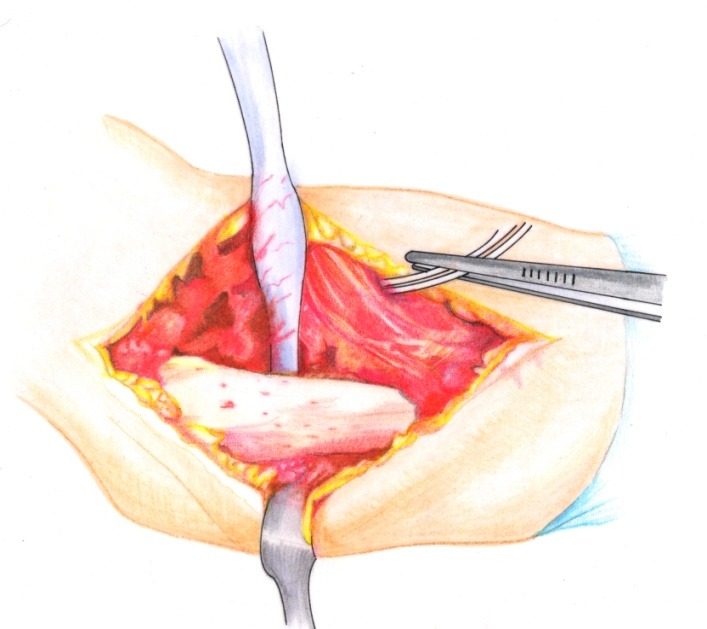
The distal diaphyseal-metaphyseal segment of the humerus after periosteum separation

**Fig. 7 a,b:**
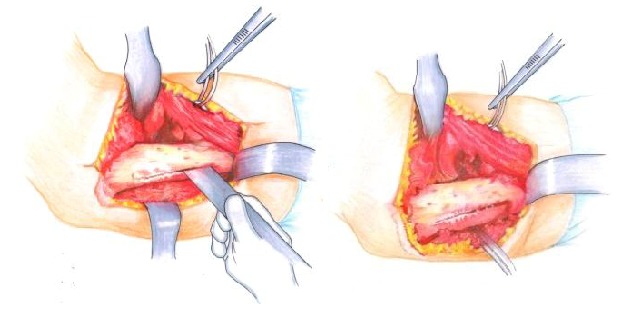
The longitudinal osteotomy must be performed using a fine blade through the middle of the bone

**Fig. 8:**
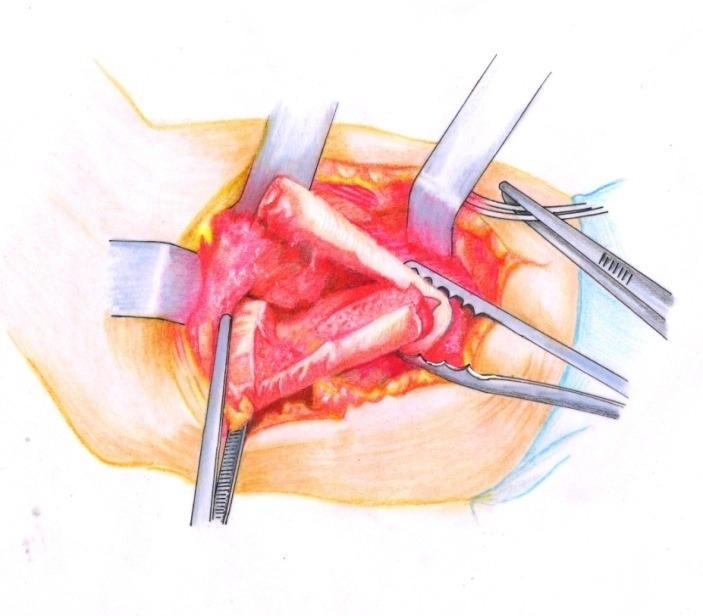
Completing the “Z” osteotomy. It is done in this manner to avoid the a hypertrophic callus that could obdurate the olecranon fossa and restrict the elbow flexion

**Fig. 9:**
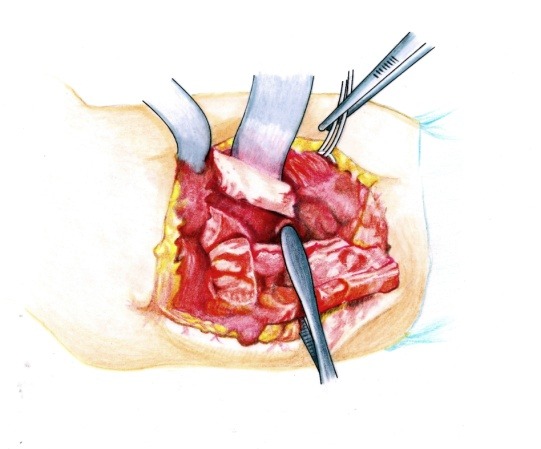
Cuneiform resections are done at the two bony extremities

**Fig. 10:**
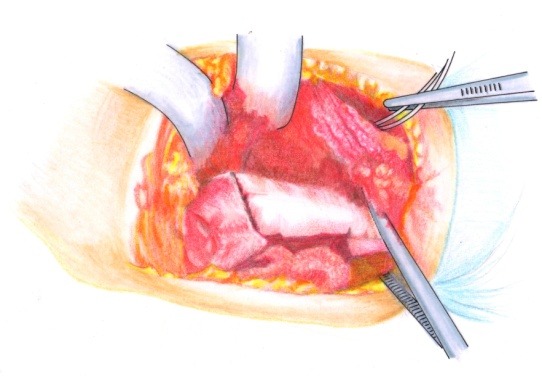
The osteoplasty appearance after physiologic centering of the thoracic limb

**Fig. 11:**
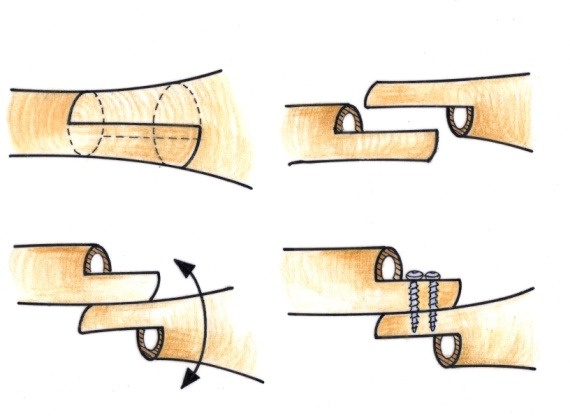
The modification of the fragments’ position according to Burnei

**Fig. 12:**
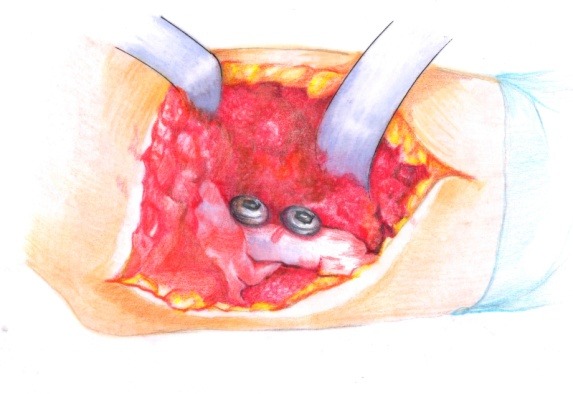
Osteotomy fixation

• A Kocher approach was performed (**Fig. 1**).

• The brachial fascia was sectioned (**Fig. 2**) and the brachial muscle with the lateral septum (**Fig. 3**) were exposed. It must be taken into account that the lateral septum can be posteriorly displaced due to malunion of the fracture site. The ulnar nerve should be isolated (**Fig. 4**).

• After the muscle release from the lateral septum, the periosteum was sectioned (**Fig. 5**).

• The distal humeral metaphyseal-diaphyseal area, was underlined by using two elevators, was enlarged and deformed (**Fig. 6**).

• By using an oscillating saw, the distal metaphyseal-diaphyseal area was sectioned in the longitudinal axis to a length of about 8 cm long (**Fig. 7 a,b**).

• The longitudinal osteotomy was transversally continued, posterior in the proximal end and anterior in the distal end (**Fig. 8**), thus obtaining the “Z” osteotomy.

• In order to achieve an osteoplastic correction, cuneiform resections were done with the base laterally oriented for varus and medially for valgus, at the ends of the two bony fragments (**Fig. 9**).

• The cuneiform fragments resected were equivalent to the axial deviation angle. The extremities were put in contact and the thoracic limb was aligned (**Fig. 10**). It must be taken into account that in children older than 10 years, the correction must be done anatomically, while in younger children, the correction must be done in a physiological manner, or even in hypercorrection. When malrotation of over 20 degrees was present, the Burnei modification had to be made: the two fragments were placed cortical to cortical in order to correct the varus/valgus as well as the rotation of the distal fragment (**Fig. 11**).

• The osteotomy site was stabilized by using two transcortical screws, introduced from anterior to posterior (**Fig. 12**).

• The planes were anatomically sutured and an intradermal suture was done, followed by dressing.

**Fig. 13:**
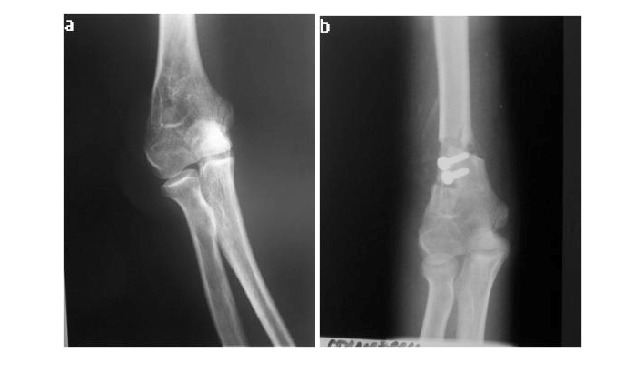
**a.** Preoperative radiologic appearance **b.** Postoperative appearance

A brachial-palmar cast immobilization was applied with the elbow in 90 degrees of flexion, for a period of 35-40 days, depending on the child’s age. An X-ray exam was done when the cast was taken off to control the consolidation (**Fig. 13 a,b**).

After the cast was taken off, a recovery treatment was recommended.

## Results

Three months past, an X-ray exam and a clinical check-up had to be performed and if a good consolidation of the osteotomy was seen, the patient was allowed to make moves that imply force. Four months postoperatively, the patient fully regained flexion and extension. Sports activities could be resumed after 6 months postoperatively.

The results were very good. In 5 cases out of the 28 (17%), an apparent residual elbow was encountered and one case of relapse (3%) was noted due to an inadequate term of cast immobilization. The elbow’s mobility was completely recovered, the thoracic member’s axis was appropriate and the metaphyseal diaphyseal osteotomy site completely healed in 3 months’ time.

## Discussions

The technique was easily performed. The correction of the deviation was easily done by placing the elbow in full extension, the fragments setting indicating the position and the dimensions of the cuneiform resections.

**Fig. 14:**
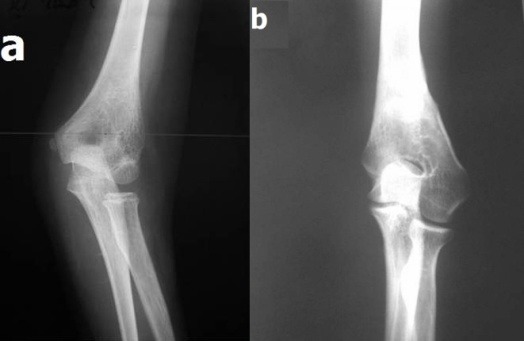
**a.** A marked deviation of the elbow that exceeds 40 degrees after a malunion of a supracondylar fracture of the humerus **b.** The axis of the thoracic limb is normal at growth end and the X-ray shows the osteoplastic effect of the intervention

Complications have arisen very rarely. Recurrence was seen when the closing cuneiform resection or the opening osteotomy did not correct the three deformities: malrotation, flexion-extension, varus-valgus. It was possible to correct the three spatial deviations by using a concept of cuneiform resection described by Takeyasu et al. [**7**], in which the resection maintained a variation in all three planes so that the correction was spatial. It is difficult to maintain a correction and to acquire the planned carrying angle because of the small area of bone contact when performing a three-dimensional osteotomy [**8**]. The Burnei modification, which offers a spatial correction by placing the elbow in full extension after the Z osteotomy is much easier and efficient and the cuneiform resections are performed while followed by cortical to cortical fixation with 2 screws. This modification allows a spatial correction, which, 3 months postoperatively, can restore the diaphyseal metaphyseal configuration of the distal humerus. Rarely, recurrence may appear when inadequate term of cast immobilization is performed. The apparent residual elbow is encountered when the rotational deformity is not corrected and when the lateral condyle is emphasized. This is an apparent deformity and that is why the possibility of cortical to cortical metaphyseal diaphyseal osteoplasty, Burnei modification, must be taken into consideration (**Fig. 14 a,b**).

**Hemiepiphysiodesis in malunited fractures** has limited indications. They can be performed only in humeral supracondylar fractures that did not affect the physis. CT and MRI are mandatory when considering this surgical technique. The physis must be free, with no bony bridges present and it must not be embedded in hypertrophic callus. This technique cannot correct malrotation. Flexion-extension deviation can be partially corrected when the “8” plate is obliquely placed. The intervention is indicated when the deviation is present only in the coronal plane.

**Zigzag indented osteotomies** described by Dobosiu were performed in “Alexandru Pesamosca” Clinique in adolescents. The bony spurs obtained after osteotomy allow the placement of the distal humeral fragment in the anatomical position, but this type of osteotomy partially corrects flexion, malrotation and sometimes valgus and varus due to secondary displacement and cast immobilization.

**Step-cut osteotomies** allow the flanking of the osteotomy by a bony spur which ensures the fixation stability and, occasionally, the fixation by using a screw that passes through the bony spur. This method ensures an enlarged contact surface of the cancellous bone, being a reasonable alternative for the correction of cubitus varus, with a satisfying deformity correction, solid healing of the osteotomy site and with a low complications rate [**9**]. This osteotomy is excellent in correcting the coronal plane deformity, offering good technical and functional results [**10**], but the risk of secondary displacement is present under the cast immobilization, especially when the edema diminishes. The osteotomy is relatively simple to be performed and has certain stability offered by the screw fixation.

**Closing wedge osteotomies** with the removal of a bone wedge from the radial side in case of cubitus varus, followed by K wire fixation or staples ensure a proper correction at the follow-up, but when the deformity was either due to physeal damage or a supracondylar fracture with secondary physeal injury, the correction was not maintained [**11**].

**Medial open wedge osteotomy with external fixation for cubitus varus deformity (Ilizarov method)** is performed by using an antero-medial approach with an exposition and anterior transposition of the ulnar nerve. Under image intensifier control, insertion of four Schanz screws is performed from medial to lateral. In between, an incomplete medial osteotomy is performed obliquely (in the antero-posterior as well as in the medio-lateral plane), leaving a small part of the radial humeral cortex intact. The osteotomy is opened until varus and, if present, hyperextension deformation is corrected. Then, the fixator system is applied [**12**]. The described technique is an alternative to other procedures, with special regard to the cosmetic outcome as well as to the control of correction for valgus and flexion [**13**].

**Supracondylar humeral osteotomies using the monoplane external fixator.** Traditional methods of correcting malunited distal humeral fractures in children involve complex wedge osteotomies held with pins or internal fixation devices. These require a large exposure and challenging fixation. Pin fixation requires extended periods of casting to protect the osteotomy until the bony union has occurred. The loss of fixation with pins is a known complication. The surgical technique consists of placing proximal and distal pin clusters consisting of 2.5 mm end-threaded AO Schanz pins before the osteotomy. The distal pin cluster consisted of two or three pins laterally placed, parallel to and 1 cm proximal to the distal humeral growth plate. The proximal pin cluster consisted of two or three pins laterally placed, in line with the humeral shaft. A small longitudinal incision was made laterally, between the pin clusters. A right-angle osteotomy was performed just above the distal pin cluster. Osteotomy stabilization was done by applying the monoplane external fixator. Initially, the malrotation was corrected, and afterwards the angulation. This technique allowed early elbow mobilization and physical therapy was recommended immediately postoperatively. The osteotomies heal at an average of 8 weeks in which time the external fixator can be removed [**14**].

**Supracondylar Humeral Osteotomies Using the Methyl Methacrylate External Fixator** [**15**]. The attempt to correct the 3 deformities present in malunited supracondylar fractures has compelled the practicing doctors not only to correct the deformities, but also to adequately stabilize the bony fragments. After the cuneiform resection is performed, the bony fragments are each fixed by using 3 to 5 pins to ensure the stability of the osteotomy site and the maintenance of the reduction is possible with polymethylmethacrylate. The pin fixation and stabilization by using methyl methacrylate ensures the correction of 2 or 3 deviations, but presents the disadvantage of destabilization once the external fixation is traumatically destroyed [**16**].

Unlike these techniques, Pesamosca osteoplasty allows a simple and efficient correction of all of the three deformities – malrotation, varus-valgus, flexion-extension.

## Conclusions

The risk of secondary displacement and surgical inefficiency is minimal, almost absent, because performing the osteoplasty in the correct manner, followed by the right positioning of the thoracic limb in his axis, with the hand in supination, disposes the fragments in the position in which the fixation must done. If a cuneiform opening is maintained, laterally or medially, the Burnei cortical-to-cortical modification must be performed. In comparison with the other surgical techniques, this technique offers the possibility to correct the valgus and varus as well as the malrotation in a simple and efficient manner.
